# Excess folic acid intake increases DNA de novo point mutations

**DOI:** 10.1038/s41421-022-00512-0

**Published:** 2023-02-28

**Authors:** Xuanye Cao, Jianfeng Xu, Ying L. Lin, Robert M. Cabrera, Qiuying Chen, Chaofan Zhang, John W. Steele, Xiao Han, Steven S. Gross, Bogdan J. Wlodarczyk, James R. Lupski, Wei Li, Hongyan Wang, Richard H. Finnell, Yunping Lei

**Affiliations:** 1 Center for Precision Environmental Health, Department of Molecular and Cellular Biology, Baylor College of Medicine, Houston, TX USA; 2 Division of Biostatistics, Dan L. Duncan Cancer and Department of Molecular and Cellular Biology, Baylor College of Medicine, Houston, TX USA; 3 Department of Pharmacology, Weill Cornell Medical College, New York, NY USA; 4 Departments of Molecular and Human Genetics, Baylor College of Medicine, One Baylor Plaza, Houston, TX USA; 5 Human Genome Sequencing Center, Baylor College of Medicine, Houston, TX USA; 6 Texas Children’s Hospital, Houston, TX USA; 7 Department of Pediatrics, Baylor College of Medicine, Houston, TX USA; 8 Department of Biological Chemistry, University of California Irvine, Irvine, CA USA; 9 Obstetrics and Gynecology Hospital, State Key Laboratory of Genetic Engineering at School of Life Sciences, Key Laboratory of Reproduction Regulation of NPFPC, Children Hospital, Fudan University, Shanghai, China

**Keywords:** Molecular biology, DNA damage and repair

Dear Editor,

It is well established that folic acid (FA) supplementation can significantly reduce the risk of birth defects, including neural tube defects (NTDs)^1^ and congenital heart defects (CHDs)^2^. More than 80 nations have adopted mandatory FA food fortification programs^3^. With additional FA intake from different dietary supplements, a portion of the population is exposed to FA concentrations over the 0.4 mg recommended daily allowance (RDA)^4^. These people include women who had a prior NTD complicated pregnancy and are planning to start a new pregnancy^5^, and men with fertility issues who are treated with high-dose FA (12.5 times of RDA) supplementation to improve sperm counts^6^. However, there is a lack of research on whether excessive FA intake has the potential to harm human beings. Recently, a case with “pseudo-MTHFR” syndrome was reported, a wild-type MTHFR Caucasian woman with a history of infertility received a high FA exposure (5 mg/day, 12.5 times of RDA), which resulted in her homocysteine levels being elevated to 17.2 μmol/L, more than double the reference homocysteine level (7.8 μmol/L). After treatment with 5-methyltetrahydrofolate (5-MTHF, 500 μg daily), her Hcy level decreased to 8.2 μmol/L, an appropriate baseline level for wild-type MTHFR individuals^7^. Other reported adverse effects of high folate intake include acute nephrotoxicity, vitamin B12 deficiency and other unwanted effects^8^. Since FA is crucial for DNA synthesis and methylation, we were concerned that excess FA administration might adversely affect the prevalence of DNA point mutations as was observed in studies of folate deficiency^9^. To this end, we sought to quantify the potential impact of FA supplementation on de novo spontaneous mutation rates and its effect on whole-genome methylation modification.

C57BL/6 J mice were maintained on three different FA diets, including FA-low (0.3 ppm FA), FA-control (3 ppm FA), and FA-high (30 ppm FA) for four months. According to the conversion of animal doses to human equivalent doses based on body surface area used in the “US FDA 2005 Guideline for Industry”, 3 ppm (control) equivalent to an average of 0.11–0.18 mg per day for an adult mouse which eats 3–5 g of mouse chow per day, about a quarter (1/4) to a half (1/2) of the US CDC RDA (0.4 mg). The 30 ppm FA mouse diet is the equivalent up to 1.8 mg FA per day, which is close to half of US CDC FA supplementation recommended dosage (4 mg) for prevention of an NTD recurrence. After these four months of treatment, female and male mice were allowed to mate with mice which were maintained on the control diet. For each treatment, either male or female mice were provided the test diet. There was no treatment group in which both parents were maintained on the test diet at the same time. A portion of the tail from both males and female parental mice along with whole embryos at E12.5 (embryo day 12.5) were collected for DNA isolation and whole genome sequencing (WGS). DNA was obtained from 77 mouse parent-embryo pairs, including 26 pairs in the 0.3 ppm FA treatment group (paternal 17, maternal 9); 26 pairs in the 3 ppm FA control group and 25 pairs in the 30 ppm treatment group (paternal 17, maternal 8). WGS with an average depth of 33× coverage was performed using the Illumina platform. After removing potential false positive signals by applying an in-house filtering algorithm, and manual bam file confirmation, we obtained 2621 de novo heterozygous single nucleotide variants (DNSNVs). Among them, 1096 DNSNVs were detected in the FA-low (0.3 ppm) group embryos (*n* = 26), 548 DNSNVs were detected in the FA-control (3 ppm) group embryos (*n* = 26), and 977 DNSNVs were identified in the FA-high (30 ppm) group embryos (*n* = 25). The median DNSNV counts per embryo in the three FA diets groups were 36 (95% CI: 24–46), 19.5 (95% CI: 14–26), and 35 (95% CI: 24–42) in FA-low, FA-control, and FA-high diet groups, respectively. The median DNSNVs per embryo for 5 subgroups were also calculated and listed as the following: paternal FA-low group: 33 (95% CI:22–46), maternal FA-low group: 38 (95% CI:26–63), control group: 19.5 (95% CI:14.0–26.0), paternal FA-high group: 40 (95% CI:26–46), maternal FA-high group: 23.5 (95% CI:15.0–78.0). The mutation rate in the three FA-supplementation groups was 1.62 × 10^–8^, 8.07 × 10^–9^, and 1.5 × 10^–8^ per nucleotide per generation, respectively. Compared to DNSNVs in the FA-control diet group, there was a significant increase in the number of DNSNVs in both FA-low groups (2.0-fold, *P* = 1.1 × 10^–4^) and FA-high groups (1.8-fold, *P* = 6.9 × 10^–4^) (Fig. 1a). Whole blood folate levels were measured using an ELISA-based methodology. Compared with the FA-control diet, the FA-high diet significantly increased whole blood folate concentrations while the FA-low diet significantly decreased whole blood folate concentrations, as would be expected (Supplementary Fig. S1).

**Fig. 1:**
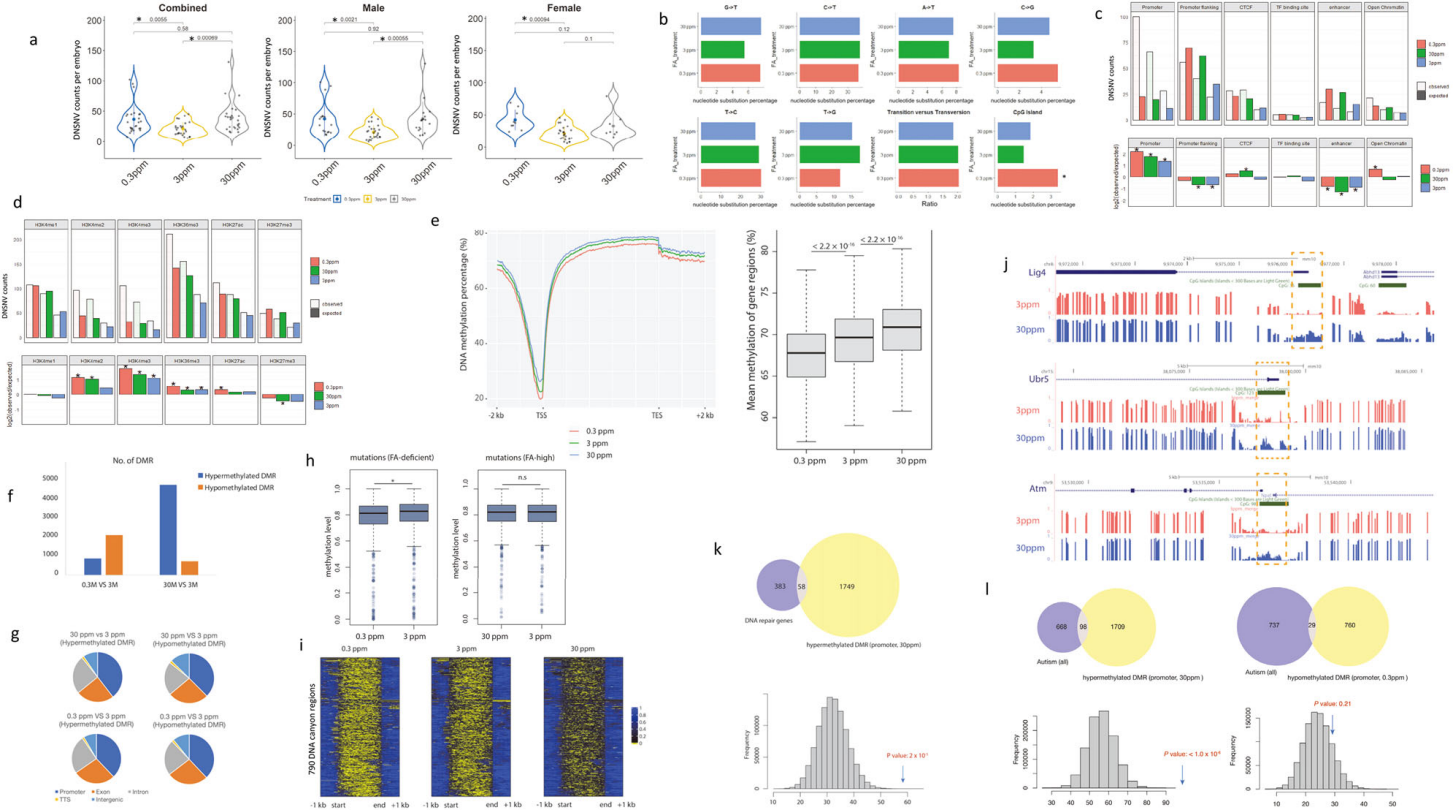
Both high FA and low FA intake increases de novo mutation rate and disrupt genome DNA methylation in the offspring. **a** Violin Plot of DNSNV/DNM (de novo mutation) counts among the three different FA dietary groups (sample size for each group was as follows; combined panel: 0.3 ppm group *n* = 26; 3 ppm group *n* = 26; 30 ppm group *n* = 25. Male panel: 0.3 ppm group *n* = 17; 3 ppm group *n* = 26; 30 ppm group *n* = 17. Female panel: 0.3 ppm group *n* = 9; 3 ppm group *n* = 26; 30 ppm group *n* = 8). Mann-Whitney *U* test was performed to test the differences among different groups. **b** Nucleotide substitution, Ts/Tv transition versus transversion ratio and CpG island DNM enrichment. Asterisks indicate significant enrichments (*P* < 0.05, one-sided binomial test). **c** DNSNV counts and enrichment analysis of promoter, promoter flanking, CTCF, TF binding site, enhancer, and open chromatin DNA region. Plots showed log2 ratio of the number of observed and expected DNSNVs indicates the effect size of the enrichment or depletion in each region. Asterisks indicate significant enrichments or depletion (*P < 0.05, one-sided binomial test). **d** DNSNV counts and enrichment analysis in histone methylation genomic DNA recognition regions, including H3K4me1, H3K4me2, H3K4me3, H3K36me3, H3K27ac and H3K27me3. Asterisks indicate significant enrichments or depletion (*P* < 0.05, one-sided binomial test). **e** Metagene plot showing the methylation level (percent methylated) of 2 kb upstream, TSS (Transcription Start Site), gene body, TES (Transcription End Site) 2-kb downstream region of all RefSeq annotated genes. The red, green, and blue lines represent FA-deficient, FA-control and FA-high groups, respectively. The mean methylation level of the gene regions for FA-deficient, normal, and FA-high group are shown by boxplot. The horizontal line of the box refers to the 25th percentiles, median and 75th percentiles, respectively. **f** Number of hypermethylated and hypomethylated DMRs identified between two FA treatment groups. **g** Annotation of DMR genomic location in different FA treated groups. TTS Transcription Terminate Site. **h** Comparison of methylation levels of 2 kb regions centered on the identified mutations (left: from FA-deficient group; right: from FA-high group) in FA-deficient and FA-control group (left) or in FA-high and FA-control group (right). **i** Heatmaps showing the methylation level of 790 canyons (identified from FA-control group) in FA-deficient, FA-control and FA-high. **j** Track figure of hypermethylation DMRs in the promoters of five DNA repair genes: *Lig4*, *Ubr5*, and *Atm*. **k** Hypermethylated DMRs in FA-high group are enriched to DNA repair genes. Permutation test was performed with the *P* value: 2 × 10^–5^. **l** Hypermethylated DMRs in FA^-^high group are enriched to autism genes. Permutation (10^-6^) *P* value is less than 1 × 10^–6.^ Autism gene list is downloaded from SAFRI database (Simons Foundation Autism Research Initiative, 11-13-2019-updated-version, total counts = 832). Fisher’s Exact test *P*-value = 6.9 × 10^–8^. *P-value is represented on each plot. *P < 0.05, ns not significant.

We analyzed the characteristics and distribution of the identified DNSNVs. The number of DNSNVs in each chromosome was correlated with chromosome size, and FA treatment does not differentially affect the DNSNV distribution at the chromosomal level (Supplementary Fig. S2a, b). We classified the DNSNVs by mutation class in each group. Overall, the nucleotide substitution frequencies for DNSNVs were dominated by C-T and T-C with a transition/transversion ratio (Ts/Tv) of ~2.0 in each treatment group. We did not see any type of nucleotide substitution significantly associated with FA treatment in our data. However, we noticed an enrichment of DNSNVs in CpG island regions, especially in the FA-deficient group (Fig. 1b). DNSNVs were annotated using Ensembl Variant Effect Predictor (VEP) toolset. On average, 93.6% of DNSNVs occurred in non-coding regions (Supplementary Fig. S2c). The MutationalPatterns package was used to evaluate the enrichment or depletion of DNSNVs in specific genomic regions. DNSNVs in all three groups were enriched in gene promoter regions (Fig. 1c). Enrichment of DNSNVs was observed in H3K4me3 and H3K36me3 regions that were positively correlated with active gene transcription in both the FA-low and the FA-high groups, but negatively correlated with repressor histone markers like H3K27me3 in the FA-high groups (Fig. 1d). DNSNVs in the FA-low group were notably enriched in the H3K4me2, H3K4me3, H3K36me3 and H3K27ac regions compared with those in the other two dietary treatment groups (Fig. 1d). H3K36me3 plays an important role in DNA mismatch repair. It recruits the mismatch recognition protein MSH6 on chromatin through direct interactions with the Msh6 PWWP domain^10^. We found that high FA decreased the H3K36me3/H3 ratio (Supplementary Fig. S3a, b), which could attribute to high FA-induced MTHFR protein reduction (Supplementary Fig. S3a, c), and S-adenosylmethionine (SAM) decreasing.

As FA plays a role in DNA methylation and DNSNV rates were most significantly affected in different paternal FA dietary treatments, whole genome bisulfite sequencing (WGBS) was used to investigate DNA methylation changes on nine embryos derived from the three paternal FA treatment groups. Compared to the FA-control group, the FA-low group embryos were primarily hypomethylated while the FA-high group embryos were globally hypermethylated (Fig. 1e). Compared to the FA-control diet group, 734 hypermethylated differentially methylated regions (DMRs) and 2127 hypomethylated DMRs were detected in the FA-low group. In contrast, 4802 hypermethylated DMRs and 904 hypomethylated DMRs were detected in the FA-high treatment group (Fig. 1f). Importantly, approximately 40% of the identified DMRs were found to be localized to gene promoter regions (Fig. 1g), suggesting that the paternal FA treatment may significantly impact gene expression. Specifically, pathway enrichment and gene ontology analysis demonstrated that the hypermethylated DMRs in the FA-high group were highly enriched in the development pathways of a multicellular organism, including neural development, neural tube closure, and heart development (Supplementary Fig. S4). This result is consistent with studies on folate status and related congenital malformations, including NTDs^1^ and CHDs^2^. We also observed the enrichment of multicellular organism development, nervous system development, and neural tube closure pathways in hypomethylated DMRs in the FA-deficient group (Supplementary Fig. S5).

It is known that aberrant DNA methylation is closely related to the occurrence of gene mutations. An interesting question is whether FA dietary treatment-induced DNA methylation change is associated with an increased mutation rate. To this end, we examined the 2-kb region centered on DNSNV sites (1 kb upstream and 1 kb downstream) and found that DNA methylation levels around the DNSNVs in the FA-low group were significantly lower than those in the FA-control group, while DNA methylation levels around the DNSNVs in the FA-high group were not significantly changed compared to those in the FA-control group (Fig. 1h). Since DNA methylation canyons (a region over 3.5 kb with less than 10% DNA methylation^11^) are highly enriched in developmental genes, such as the homeobox genes, we examined 790 canyon regions identified in the FA-control group embryos. The methylation levels of these canyons were increased in the high FA treatment groups and decreased in the low FA group, respectively (Fig. 1i). There was no difference in the distribution of observed DNSNVs in the canyon regions in FA-high group (2 out of 978 DNSNVs in canyon region, *P* = 0.39) and in FA-control group (1 out of 546 DNSNVs in canyon region, *P* = 0.2) compared with theoretical expectations. However, the number of DNSNVs observed in canyon region in FA-low group (12 out of 1097 DNSNVs in canyon region) was significantly higher than expectation (*P* = 1.6 × 10^–4^).

It is well established that the gene-promoter-region methylation level is negatively associated with gene expression, while the gene-body-region methylation level is positively associated with gene expression^12^. We paid particular attention to whether promoter hypermethylated DMRs enriched in DNA repair genes in the FA-high group, as the mutation rates would be increased secondary to the reduced activity of DNA repair pathways. Hypermethylated DMRs were observed in promoter regions of 58 DNA repair genes (Supplementary Table S1), including *Lig4*, *Ubr5*, and *Atm* (Fig. 1j). Compared to all genes with hypermethylated DMRs in the promoter region, there was a significant enrichment of promoter hypermethylated DMRs in DNA repair genes (*P* = 2 × 10^–5^) (Fig. 1k). Taken together, both low and high intake of FA altered the DNA methylation landscape and increased mutation rates. However, the mechanism by which methylation disruption increased the mutation rate between the FA-low and the FA-high groups may be different. While the FA-low diet increased mutation rate through deaminating methylated cytosine (5mC) at CpG sites to thymine, the FA-high diet could increase mutation rate through down-regulation of DNA repair pathways by hypermethylation promoter CpG island.

A recent study suggested that high FA exposure may be associated with an increased risk for autism spectrum disorders (ASD)^13^. We examined FA dietary effects on methylation regulation of 766 reported ASD genes (SFARI, 2019) and identified significant enrichment of hypermethylated DMRs in the promoter regions of ASD genes in the FA-high group (Fisher’s exact test *P* = 6.9 × 10^–8^), but not in the FA-low group (Fig. 1l). It is known that both DNSNVs and DNA methylation play important roles in the etiology of ASD. In fact, 15%–30% of ASDs are thought to result from deleterious DNSNVs, and there is increasing evidence for methylation-dependent regulation of transcription as a causative factor in the etiology of ASD^14^. Our results indicate that excessive parental FA intake in mice can result in promoter hypermethylation of some human candidate ASD genes in mouse embryos at E12.5. Further studies are required to test the link between hypermethylation and gene expression at different time points.

This study was designed to query for the first time the effect on DNA mutation rate using WGS and WGBS from mice exposed to different FA diets. We found that an FA-high diet significantly increased DNA de novo point mutation frequency, as was the case in the FA-low diet. Compared to the FA-control group, the median number of DNSNVs per embryo increased 2-fold in the FA-low group and 1.8-fold in the FA-high group. There were sex differences in the FA dietary treatment responses based on the median DNSNV number. Male mice were more sensitive to the FA-high treatment compared to female mice. This difference may relate to gender differences in germline stem cell proliferation, as male mice keep generating new sperm. In contrast, female oocytes do not increase much after birth. The treatment of FA was initiated after weaning, which may affect sperm de novo mutation rates that are more profound than what could be observed in the oocytes. Another explanation is that higher resorption rates in the FA-high female group (FA-high: 16 resorption and 76 live embryos vs. FA-control: 3 resorption and 91 live embryos, Fisher Exact test *P* = 0.027) may eliminate embryos with higher mutation rates which could skewed the results in a downward direction. WGBS demonstrated that DNA repair genes were significantly hypermethylated in the FA-high diet, indicating that excess folic acid supplementation may affect the de novo mutation rate through downregulating DNA repair genes expression and consequently impaired DNA repair activity. A recent study also suggested that excess folic acid concentrations can cause DNA damage induced by oxidative stress and DNA base excision repair gene expression^15^. The mechanisms by which DNA repair genes are significantly hypermethylated in FA-high group are not fully understood and require further investigation. Our data indicate that FA supplementation should be restricted to an ideal benefit range. Both insufficient folate and excess FA intake are risk factors for genome and epigenome instability. The genome and epigenome impairment induced by either high FA or low FA diets should be further confirmed in human population in future studies.

## Supplementary information

Supplementary Information
