# Supplementary material for: Excess folic acid intake increases DNA de novo point mutations
Source: Cell Discov. 2023 Feb 28;9:22. doi: 10.1038/s41421-022-00512-0 (PMC9970956; doi:10.1038/s41421-022-00512-0)
Supplement: Supplementary file 1 — Supplementary Information [file 41421_2022_512_MOESM1_ESM.pdf]

Material and Methods

Mouse Cohort Description and Dietary Intervention

All experiments utilizing animals were approved by the Baylor College of Medicine Institutional Animal Care and Use Committee. C57BL/6J mice were obtained from The Jackson Laboratory (Bar Harbor, ME). Clifford/Koury folate deficient L-amino acid defined rodent diets (Diet # 517777) with conventional (3mg/kg, Diet # 517940), deficient (0.3mg/kg, Diet # 517796), and excess FA (30mg/kg, Diet # 517941) content with 1% succinyl sulfathiazole to inhibit FA synthesis by the intestinal flora were purchased from Dyets, Inc. (Bethlehem, PA).

Female mouse treatment groups

Virgin females 8 weeks of age were randomly assigned to receive either a control diet containing 3 mg FA/kg diet or a FA-deficient diet containing 0.3 mg FA/kg diet, or a FA-supplemented diet containing 30 mg FA/kg for four months prior to the first attempt to mate with mice which were kept on the control diet. Each time, only one of the parents was on testing diet. Female mice were continually maintained on the testing diets until the time the dams were euthanized on E12.5 to harvest embryos.

Male mouse treatment groups

Virgin males 8 weeks of age were randomly assigned to receive either a control diet containing 3 mg FA/kg diet or a FA-deficient diet containing 0.3 mg FA/kg diet, or a FA-supplemented diet containing 30 mg FA/kg for four months prior to the first attempt at mating. Embryos were harvested at E12.5 from pregnant females.

Blood folate measurement

Blood folate levels were measured using a folate competitive analysis as previously reported[1]. Briefly, FOLR1 (1 µL at 50ng/µL) was immobilized on microtiter plates in PBS buffer (pH 8.0). The following day, unbound protein was removed by washing three times with tris-buffered saline (pH 7.6). Ligand in the form of FA is used to compete with labeled (e.g., horse radish peroxidase) folic acid (FA-HRP) for competitive binding to FOLR1. Standard dilution (1:2) of FA (6 to 0.8125ng/mL, 0ng/mL) results in FOLR1-folate binding curves.

Whole Genome Sequencing (WGS)

DNA extraction

Mouse embryos and parental tail tissues were collected and maintained at -20˚C. Qiagen QIAamp DNA micro-Kit (Cat. No. 56304) was used for DNA extraction with RNase treatment. Qubit assays were performed to validate that all DNA samples met the quality requirements for following sequencing projects.

WGS Data Processing

Extracted genomic DNA from mouse tails or embryos were subjected to WGS on an Illumina NovaSeq. WGS raw FASTQ data (sequencing projects were conducted by Admera Health, LLC) were processed using a pipeline bas ed on GATK Best Practice. Sequence reads were mapped to the reference genome (mm10) with BWA-MEM. After sorting by Samtools, BAM files are further processed with GATK Best Practice workflows, including marking duplicates, realignment of indels, base quality recalibration, as described[2]. Single nucleotide variants and small indels were called with GATK haplotypeCaller.

DNM Calling and Variant Filtering

DNMs were called by TrioDenovo program[3]. An in-software pipeline for filtering DNSNV was used (https://genome.sph.umich.edu/wiki/Triodenovo). We followed the previously well-described filtering pipeline for the DNM filtering[4]. The hard filters include: (i) an in-cohort MAF = 0; (ii) a minimum 20 total reads, 5 alternate allele reads, and a minimum 20% alternate allele ratio in the embryo if alternate allele reads are greater than 10. If alternate reads are less than 10, a minimum 30% ratio is required. (iii) a minimum depth of 15 reference reads and alternative allele < 3.5% in the parents. All the DNMs were annotated by VEP (Ensembl Variant Effect Predictor).

Quality control and validation

All the DNSNVs are then filtered from 'Bamfile' confirmation and filtered with a lowest genotype quality (Qual) > 50. We extracted all of the 'Bamfile' containing the region of each DNSNV and its surrounding 2000bp. We examined each mutation in the three lanes of 'Bamfile' checking process via using Integrative Genomics Viewer. The filters are (i) minimum depth of 15 reference reads and alternative allele < 3.5% in the parents; (ii) mutations in the same read are excluded if it is in the same haplotype. To estimate the true positive rate of our DNSNV dataset, we successfully sequenced 72 DNSNVs, and 70 out of 72 were confirmed by Sanger sequencing (True Positive Rate = 97.2%). Finally, we performed a validation test on our mutation calling pipeline using 'Genome in a bottle' as a reference. Using our pipeline, we obtained a sensitivity of 1039 / (1039 + 5) = 0.9952107, and a specificity of (4121168 - 1044 - 1) / (4121168 - 1044) = 0.9999998.

Whole genome bisulfide sequencing

WGBS data processing

Raw bisulfite sequencing data of mouse embryos is mapped to mm10 mouse reference genome by BSMAP (2.90) with default setting. The contaminated adapters and low-quality bases are removed

with the '-q' and '-A' parameters during alignment. We subsequently used the methratio.py script provided by BSMAP to calculate the methylation levels of CpG sites. CpG sites that are covered less than 4 reads are discarded before the downstream analysis.

De novo differentially methylated regions (DMRs) and DNA methylation canyon calling

We identify *de novo* DMRs between two conditions with the '*de novo'* mode of Metilene (0.2-7)[5]. DMRs with mean methylation change greater than 0.2 and q value less than 0.05 are selected for downstream analysis. The identified DMRs are annotated to the nearest gene. KEGG pathway enrichment and gene ontology analyses were performed by using DAVID. For DNA methylation canyon identification, we used the method described by Mira Jeong[6]. Boxplots were used to depict the mean methylation level of the gene regions for FA-deficient, normal, and FA-high groups. The horizontal line of the box refers to the 25th percentiles, median and 75th percentiles, respectively. Whiskers represents the 1.5X interquartile range. Student's t-test was performed to compare the mean methylation level of gene regions for different FA treatment groups.

Western blotting

Proteins were extracted from NE-4C cells cultured for 72 h in FA control (0.5µM) or FA high (5µM) medium. Antibodies: anti-MTHFR (1:1000, Cat#: ab203786, Abcam, Cambridge, MA, USA), anti-GAPDH (Cat#:5174S, Cell Signaling Technology, Inc, Danvers, MA), anti-H3K36me3 (1:1000, Cat #9763, CST, MA, USA), Anti-Histone H3 (1:10000, ab18521, Abcam, Cambridge, MA, USA) were used. GAPDH and H3 were references for MTHFR and H3K36me3 relative calculation respectively.

Reverse transcription-quantitative polymerase chain reaction (RT-qPCR)

mRNA was extracted from mice embryos using quick-RNA Tissue/Insect Kit (Cat#:R2030, Zymo Research, Irvine, CA, USA). RNA quantification was measured using Nanodrop 200. RT-qPCR was performed with One Step TB Green PrimerScript RT-PCR kit II (Perfect Real Time) from TakaRa (Cat#RR086A) following the product instruction. PCR was performed on a Bio-Rad CFX96 Optics Module qPCR/RT PCR Thermal Cycler. Primers used were as following: Lig4rtF:CTAGCTACCTGGGACCTGCG; Lig4rtR:GCAAAGGGGACATGAGCTGC; Ubr5rtF:GCTATTGCGGTCAGGGAGC; Ubr5rtR:CATGGTGATGGGACGGCC; AtmrtF:AGGCATTCTGATTCCAAACAAGG; AtmrtR:TCTGGAGCTCTGTGTGGTGG; GapdhrtF:CTTCTTGTGCAGTGCCAGCC; GapdhrtR:TGAAGGGGTCGTTGATGGCA.

Statistical analysis

Differences in DNSNV counts among groups were examined with Wilcoxon signed-rank test or Student's t-test. Fisher's exact test was used to examine the enrichment among different groups. Statistical analyses were conducted using R Studio 3.5.2. A two-tailed *P*-value of <0.05 was considered statistical significance. *P*-adj values were all calculated by FDR correction.

Data access

The raw sequencing data that support the findings of this study were submitted to NIH SRA database (BioProject ID: PRJNA793863 for WGS data and PRJNA791955 for WGBS data).

References:

1 Cabrera RM, Souder JP, Steele JW *et al.* The antagonism of folate receptor by dolutegravir: developmental toxicity reduction by supplemental folic acid. *AIDS* 2019; **33**:1967-1976.
2 McKenna A, Hanna M, Banks E *et al.* The Genome Analysis Toolkit: a MapReduce framework for analyzing next-generation DNA sequencing data. *Genome Res* 2010; **20**:1297-1303.
3 Wei Q, Zhan X, Zhong X *et al.* A Bayesian framework for de novo mutation calling in parents-offspring trios. *Bioinformatics* 2015; **31**:1375-1381.

4 Jin SC, Homsy J, Zaidi S *et al.* Contribution of rare inherited and de novo variants in 2,871 congenital heart disease probands. *Nat Genet* 2017; **49**:1593-1601.
5 Jühling F, Kretzmer H, Bernhart SH, Otto C, Stadler PF, Hoffmann S. metilene: fast and sensitive calling of differentially methylated regions from bisulfite sequencing data. *Genome Res* 2016; **26**:256-262.
6 Jeong M, Sun D, Luo M *et al.* Large conserved domains of low DNA methylation maintained by Dnmt3a. *Nature genetics* 2014; **46**:17.

Supplementary Figure Legends

Supplementary Fig. S1| Blood folate concentrations. Blood folate measurement showed maternal FA deficient diet decreased the blood folate level, while FA high diet increased the blood folate level significantly. One-way ANOVA with Dunnett's multiple comparisons was performed to examine whether the differences are significant.

Supplementary Fig. S2| Overall characterization of DNM distribution and location. a, Chromosome size and DNSNV distribution. Linear Regression p-value < 0.0001. Unit = base pair (bp) b, Genome-wide rainfall plot of DNSNVs of each FA-treated group. d, VEP annotation of DNSNVs from different FA treatment in mm10 genome.

Supplementary Fig. S3 MTHFR and H3K36Me3 relative protein expression in NE4C cells cultured in FA-control and FA-high medium. a, immunoblotting detection of MTHFR, GAPDH, H3K36Me3, and histone H3. b, GAPDH relative amount quantification using GAPDH as reference. c, H3K36Me3 relative amount quantification using histone H3 as reference.

Supplementary Fig. S4| Pathway enrichment of hypomethylated DMRs in FA-deficient group. Both KEGG and Gene Ontology Biological Process pathways are displayed.

Supplementary Fig. S5| Pathway enrichment of hypermethylated DMRs in FA-high group. Both KEGG and Gene Ontology Biological Process pathways are displayed. Pathway enrichment of hypomethylated DMRs in FA-high group was also shown at the right panel.

Supplementary Fig. S1

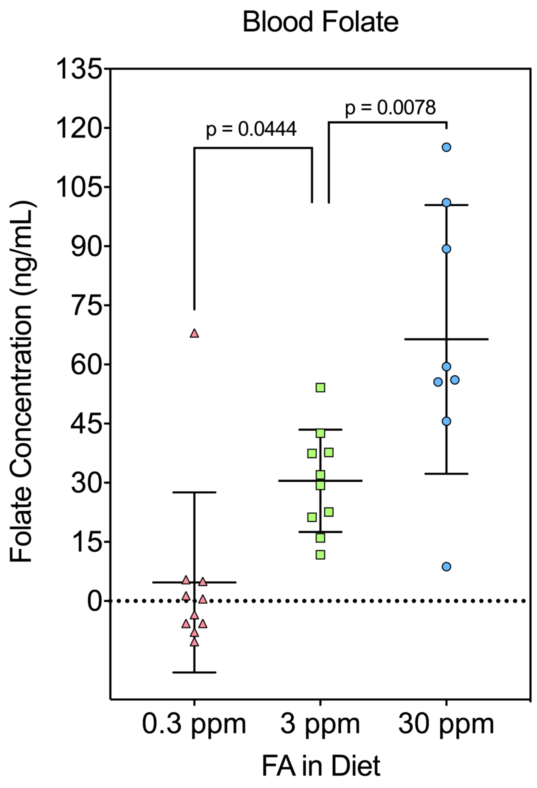

Supplementary Fig. S2

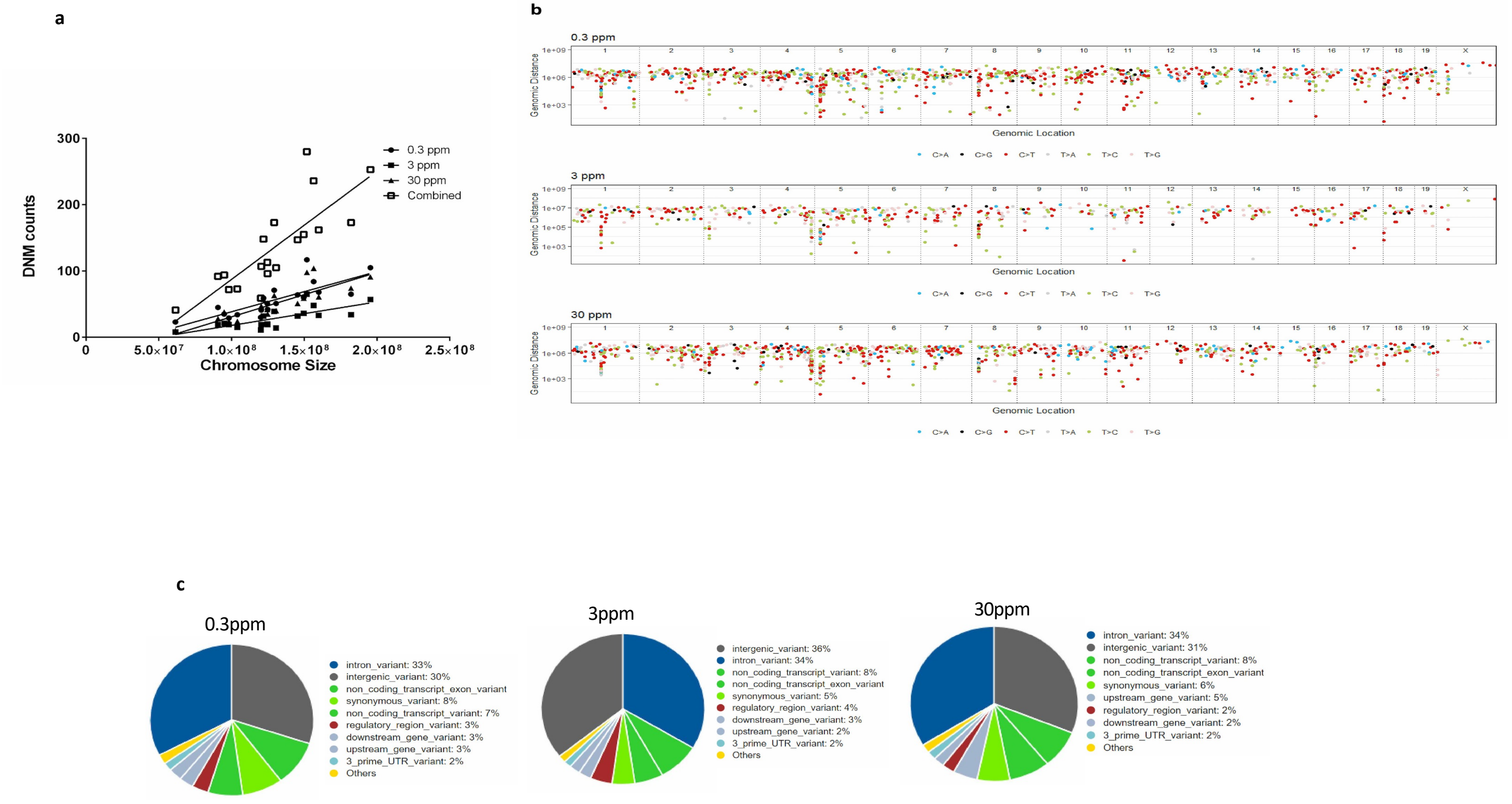

Supplementary Fig. S3

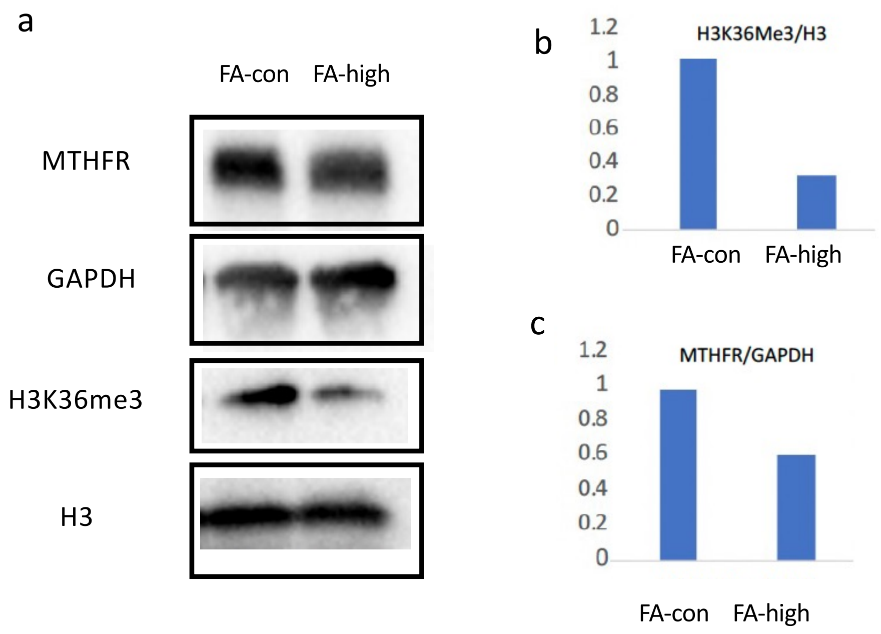

Supplementary Fig. S4

Pathway enrichment of hypermethylated DMRs in FA-high group

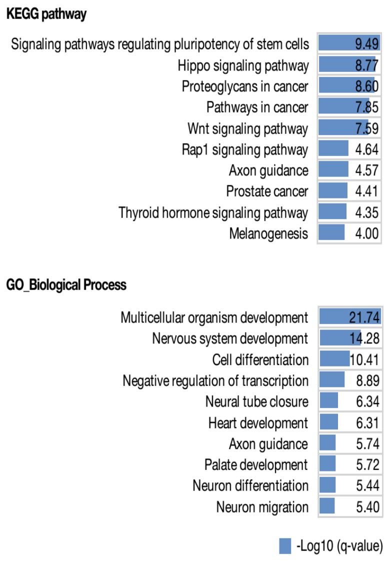

Pathway enrichment of hypomethylated DMRs in FA-high group

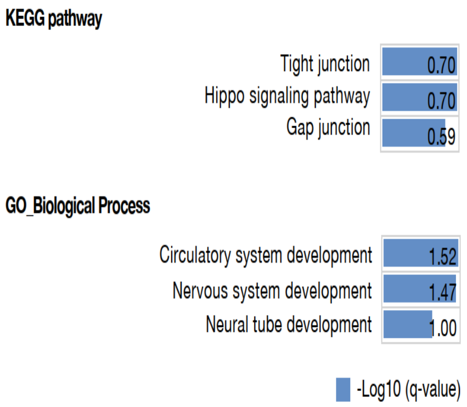

Supplementary Fig. S5

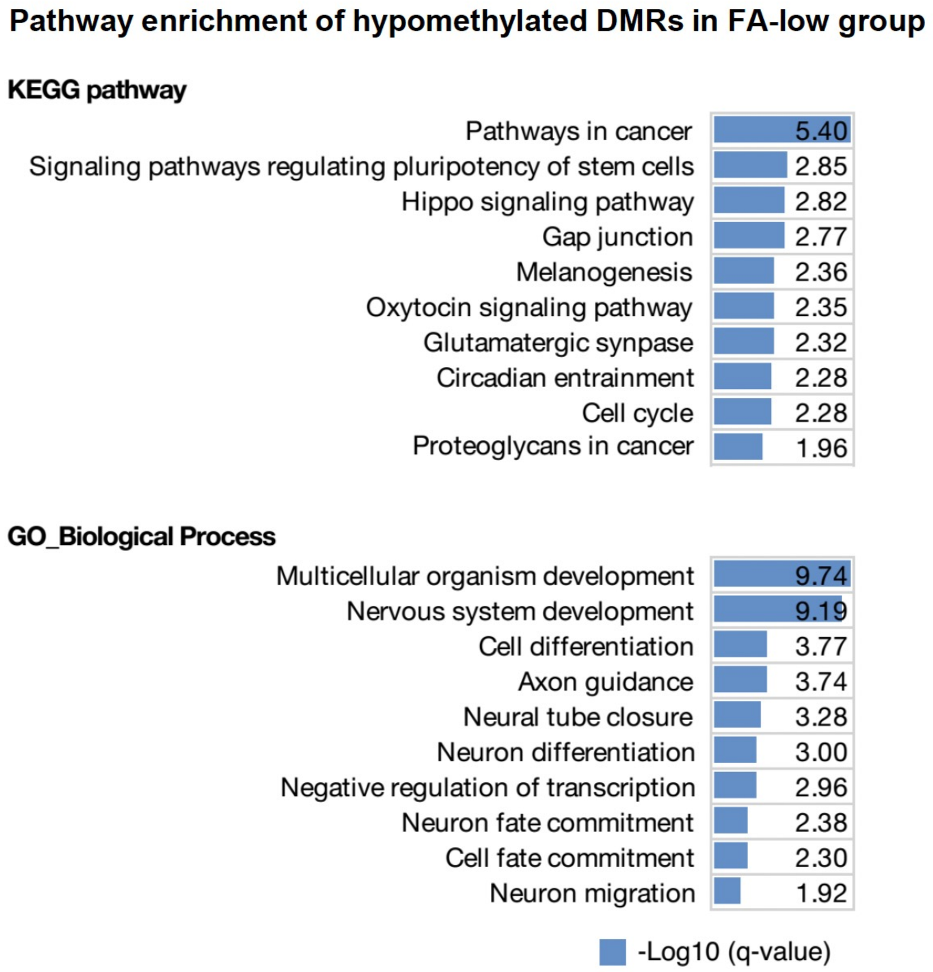

Supplement Table S1. 58 DNA repair genes with hypermethylated DMRs in the promoter

Chr	Start	End	meth_diff	q_value	Annotation	Detailed.Annotation	Distance.to.TSS	Nearest.PromoterID	Entrez.ID	Gene.Name	Gene.Description	Gene.Type	gene
chr1	191574849	191575040	0.245	0.000031	promoter-TSS (NM_001306203)	promoter-TSS (NM_001306204)	599	NM_001305233	76843	Dtl	denticleless homolog (Drosophila)	protein-coding	DTL
chr10	11149374	11149484	0.662	0.000018	promoter-TSS (NM_172937)	promoter-TSS (NM_001077707)	-1	NM_001077707	268281	Shprh	SNF2 histone linker PHD RING helicase	protein-coding	SHPRH
chr10	81378275	81378347	0.205	0.000036	promoter-TSS (NM_019757)	promoter-TSS (NM_019757)	59	NM_019757	56371	Fzr1	fizzy/cell division cycle 20 related 1 (Drosophila)	protein-coding	FZR1
chr11	4637689	4637835	0.280	0.024	promoter-TSS (NM_029291)	promoter-TSS (NM_029291)	-31	NM_029291	75452	Ascc2	activating signal cointegrator 1 complex subunit 2	protein-coding	ASCC2
chr11	5878084	5878242	0.293	0.000003	promoter-TSS (NM_008894)	promoter-TSS (NM_008894)	93	NM_008894	18972	Pold2	polymerase (DNA directed), delta 2, regulatory subunit	protein-coding	POLD2
chr11	26386293	26387370	0.214	0.01	promoter-TSS (NR_102382)	promoter-TSS (NM_025923)	-252	NM_025923	67030	Fancl	Fanconi anemia, complementation group L	protein-coding	FANCL
chr11	77607912	77608152	0.287	6.4E-18	promoter-TSS (NM_144825)	promoter-TSS (NM_144825)	-217	NM_144825	216965	Taok1	TAO kinase 1	protein-coding	TAOK1
chr11	106779472	106779631	0.369	0.000016	promoter-TSS (NR_027785)	promoter-TSS (NR_027785)	-15	NM_015810	50776	Polg2	polymerase (DNA directed), gamma 2, accessory subunit	protein-coding	POLG2
chr11	119041189	119041305	0.233	0.0064	promoter-TSS (NM_013926)	promoter-TSS (NM_013926)	-334	NM_013926	30951	Cbx8	chromobox 8	protein-coding	CBX8
chr11	119491356	119491476	0.229	1.4E-07	promoter-TSS (NM_177394)	promoter-TSS (NM_177394)	69	NM_001164636	338371	Endov	endonuclease V	protein-coding	ENDOV
chr12	11265953	11266028	0.307	0.00066	promoter-TSS (NM_177331)	promoter-TSS (NM_177331)	105	NM_025695	67241	Smc6	structural maintenance of chromosomes 6	protein-coding	SMC6
chr12	110888600	110888852	0.210	0.0063	promoter-TSS (NM_175336)	promoter-TSS (NM_175336)	419	NM_027223	67236	Cinp	cyclin-dependent kinase 2 interacting protein	protein-coding	CINP
chr12	111813613	111813729	0.218	0.028	promoter-TSS (NM_026752)	promoter-TSS (NM_026752)	170	NM_028875	74335	Xrcc3	X-ray repair complementing defective repair in Chinese hamster cells 3	protein-coding	XRCC3
chr13	32801696	32801858	0.280	0.000016	promoter-TSS (NM_030215)	promoter-TSS (NM_030215)	-253	NM_030215	78903	Wrnip1	Werner helicase interacting protein 1	protein-coding	WRNIP1
chr13	47106392	47106497	0.405	0.000032	promoter-TSS (NM_025900)	promoter-TSS (NM_025900)	-224	NM_025900	110052	Dek	DEK oncogene (DNA binding)	protein-coding	DEK
chr13	63431597	63431732	0.295	9E-09	promoter-TSS (NM_001042673)	promoter-TSS (NM_001042673)	80	NM_007985	14088	Fancc	Fanconi anemia, complementation group C	protein-coding	FANCC
chr13	92355035	92355135	0.414	0.0001	promoter-TSS (NM_010829)	promoter-TSS (NM_010829)	-82	NM_010829	17686	Msh3	mutS homolog 3 (E. coli)	protein-coding	MSH3
chr14	16364697	16365045	0.269	0.0076	promoter-TSS (NM_009409)	promoter-TSS (NM_009409)	-335	NM_009409	21974	Top2b	topoisomerase (DNA) II beta	protein-coding	TOP2B
chr14	32202276	32202429	0.378	1.1E-08	promoter-TSS (NM_016897)	promoter-TSS (NM_016897)	382	NM_011960	26430	Parg	poly (ADP-ribose) glycohydrolase	protein-coding	PARG
chr14	52197190	52197584	0.223	1E-16	promoter-TSS (NM_033618)	promoter-TSS (NM_033618)	-148	NM_033618	114741	Supt16	suppressor of Ty 16	protein-coding	SUPT16
chr14	63193795	63194047	0.262	0.00012	promoter-TSS (NM_201610)	promoter-TSS (NM_201610)	-396	NM_201610	382913	Neil2	nei like 2 (E. coli)	protein-coding	NEIL2
chr15	38078952	38079021	0.457	0.00025	promoter-TSS (NM_001081359)	promoter-TSS (NM_001112721)	-133	NM_001081359	70790	Ubr5	ubiquitin protein ligase E3 component n-recognin 5	protein-coding	UBR5
chr15	51991754	51991932	0.319	2.9E-06	promoter-TSS (NM_009009)	promoter-TSS (NM_009009)	-83	NM_009009	19357	Rad21	RAD21 homolog (S. pombe)	protein-coding	RAD21
chr15	76478108	76478325	0.237	0.0028	promoter-TSS (NM_013481)	promoter-TSS (NM_013481)	772	NM_008296	15499	Hsf1	heat shock factor 1	protein-coding	HSF1
chr16	15637198	15637429	0.214	2.3E-09	promoter-TSS (NM_011159)	promoter-TSS (NM_011159)	86	NM_008565	17217	Mcm4	minichromosome maintenance deficient 4 homolog (S. cerevisiae)	protein-coding	MCM4
chr17	27555937	27556185	0.222	2.1E-12	promoter-TSS (NM_001166544)	promoter-TSS (NM_001039356)	-513	NM_001166536	15361	Hmga1	high mobility group AT-hook 1	protein-coding	HMGA1
chr17	29614768	29614853	0.204	0.0013	promoter-TSS (NR_132314)	promoter-TSS (NR_132314)	22	NM_021419	58230	Rnf8	ring finger protein 8	protein-coding	RNF8
chr17	35821008	35821709	0.218	7.4E-08	promoter-TSS (NM_133662)	promoter-TSS (NM_133662)	-354	NM_133662	15937	Ier3	immediate early response 3	protein-coding	IER3
chr18	21001695	21001985	0.264	3.2E-08	promoter-TSS (NM_001303011)	promoter-TSS (NM_001303011)	-117	NM_001303011	56515	Rnf138	ring finger protein 138	protein-coding	RNF138
chr19	5609590	5609755	0.213	0.000085	promoter-TSS (NR_037603)	promoter-TSS (NR_037603)	34	NR_037603	81601	Kat5	K(lysine) acetyltransferase 5	protein-coding	KAT5
chr19	7206127	7206314	0.409	9.8E-08	promoter-TSS (NM_134150)	promoter-TSS (NM_134150)	63	NM_134150	107260	Otub1	OTU domain, ubiquitin aldehyde binding 1	protein-coding	OTUB1
chr19	10203879	10203952	0.277	0.000014	promoter-TSS (NM_007999)	promoter-TSS (NM_007999)	27	NM_007999	14156	Fen1	flap structure specific endonuclease 1	protein-coding	FEN1
chr19	10605446	10605677	0.346	4.7E-10	promoter-TSS (NM_015735)	promoter-TSS (NM_015735)	-63	NM_015735	13194	Ddb1	damage specific DNA binding protein 1	protein-coding	DDB1
chr2	49451039	49451162	0.220	4.6E-10	promoter-TSS (NM_172663)	promoter-TSS (NM_172663)	-385	NM_172663	227867	Epc2	enhancer of polycomb homolog 2 (Drosophila)	protein-coding	EPC2
chr2	119477703	119477782	0.240	0.00023	promoter-TSS (NM_026574)	promoter-TSS (NM_026574)	-114	NM_026574	68142	Ino80	INO80 homolog (S. cerevisiae)	protein-coding	INO80
chr2	144270861	144270986	0.235	0.0071	promoter-TSS (NM_001199188)	promoter-TSS (NM_001289631)	20	NM_001289630	74528	Mgme1	mitochondrial genome maintainance exonuclease 1	protein-coding	MGME1
chr2	147013052	147013155	0.293	0.0018	promoter-TSS (NM_011917)	promoter-TSS (NM_011917)	44	NM_011917	24128	Xrn2	5'-3' exoribonuclease 2	protein-coding	XRN2
chr2	167632073	167632271	0.376	3.2E-12	promoter-TSS (NM_023230)	promoter-TSS (NM_023230)	-167	NM_023230	66589	Ube2v1	ubiquitin-conjugating enzyme E2 variant 1	protein-coding	UBE2V1
chr3	90433519	90433571	0.214	0.0029	promoter-TSS (NM_178876)	promoter-TSS (NM_178876)	91	NM_145540	229543	Ints3	integrator complex subunit 3	protein-coding	INTS3
chr3	135438685	135439016	0.276	2.2E-09	promoter-TSS (NM_025356)	promoter-TSS (NM_025356)	92	NM_025356	66105	Ube2d3	ubiquitin-conjugating enzyme E2D 3	protein-coding	UBE2D3
chr4	21776022	21776570	0.208	5.5E-11	promoter-TSS (NM_001290425)	promoter-TSS (NM_001290425)	26	NM_152825	77593	Usp45	ubiquitin specific petidase 45	protein-coding	USP45
chr4	120924849	120925116	0.250	0.048	promoter-TSS (NM_001160043)	promoter-TSS (NM_028457)	22	NM_028457	73172	Exo5	exonuclease 5	protein-coding	EXO5
chr4	132639019	132639084	0.410	0.000088	promoter-TSS (NM_010166)	promoter-TSS (NM_010166)	6	NM_010166	14050	Eya3	eyes absent 3 homolog (Drosophila)	protein-coding	EYA3
chr5	33378516	33378563	0.317	0.000017	promoter-TSS (NM_001081101)	promoter-TSS (NM_001081101)	-156	NM_001081101	71101	Uvssa	UV stimulated scaffold protein A	protein-coding	UVSSA
chr5	65335710	65335798	0.314	0.0026	promoter-TSS (NM_011258)	promoter-TSS (NM_011258)	-115	NM_011258	19687	Rfc1	replication factor C (activator 1) 1	protein-coding	RFC1
chr5	114128110	114128499	0.258	1.5E-10	promoter-TSS (NM_175016)	promoter-TSS (NM_175016)	-129	NM_175016	231642	Alkbh2	alkB, alkylation repair homolog 2 (E. coli)	protein-coding	ALKBH2
chr5	144768725	144768858	0.292	1.7E-08	promoter-TSS (NM_001081362)	promoter-TSS (NM_001081362)	0	NM_001081362	100683	Trrap	transformation/transcription domain-associated protein	protein-coding	TRRAP
chr6	88898667	88898735	0.341	0.014	promoter-TSS (NM_008564)	promoter-TSS (NM_008564)	79	NM_008564	17216	Mcm2	minichromosome maintenance deficient 2 mitotin (S. cerevisiae)	protein-coding	MCM2
chr6	91473046	91473178	0.326	0.012	promoter-TSS (NM_028766)	promoter-TSS (NM_028766)	311	NM_133928	72170	Chchd4	coiled-coil-helix-coiled-coil-helix domain containing 4	protein-coding	CHCHD4
chr7	19345006	19345284	0.323	0.04	promoter-TSS (NM_007948)	promoter-TSS (NM_001127324)	74	NM_007948	13870	Ercc1	excision repair cross-complementing rodent repair deficiency, complem	protein-coding	ERCC1
chr7	19381859	19381977	0.217	0.000082	promoter-TSS (NM_007949)	promoter-TSS (NM_007949)	-121	NM_007949	13871	Ercc2	excision repair cross-complementing rodent repair deficiency, complem	protein-coding	ERCC2
chr7	51862284	51862560	0.334	1.9E-09	promoter-TSS (NM_001115087)	promoter-TSS (NM_001115087)	-155	NM_001115087	100040608	Fancf	Fanconi anemia, complementation group F	protein-coding	FANCF
chr7	126695373	126695539	0.252	1.1E-07	promoter-TSS (NM_175103)	promoter-TSS (NM_175103)	327	NM_029420	75764	Slx1b	SLX1 structure-specific endonuclease subunit homolog B (S. cerevisiae)	protein-coding	SLX1B
chr8	9976410	9976521	0.248	2.7E-06	promoter-TSS (NM_176953)	promoter-TSS (NM_176953)	-143	NM_176953	319583	Lig4	ligase IV, DNA, ATP-dependent	protein-coding	LIG4
chr8	22653353	22653504	0.414	4.2E-07	promoter-TSS (NM_011130)	promoter-TSS (NM_011130)	8	NM_011130	18970	Polb	polymerase (DNA directed), beta	protein-coding	POLB
chr9	31386174	31386307	0.438	2.6E-06	promoter-TSS (NM_172766)	promoter-TSS (NM_172766)	49	NM_172766	235134	Nfrkb	nuclear factor related to kappa B binding protein	protein-coding	NFRKB
chr9	44305828	44305941	0.307	0.000094	promoter-TSS (NM_172162)	promoter-TSS (NM_172162)	-214	NM_172162	102423	Hinfp	histone H4 transcription factor	protein-coding	HINFP
chr9	53536725	53536945	0.233	9.4E-06	promoter-TSS (NM_001081152)	promoter-TSS (NM_001081152)	-164	NM_007499	11920	Atm	ataxia telangiectasia mutated	protein-coding	ATM
